# Umbilical Cord Tissue as a Source of Young Cells for the Derivation of Induced Pluripotent Stem Cells Using Non-Integrating Episomal Vectors and Feeder-Free Conditions

**DOI:** 10.3390/cells10010049

**Published:** 2020-12-31

**Authors:** Aisha Mohamed, Theresa Chow, Jennifer Whiteley, Amanda Fantin, Kersti Sorra, Ryan Hicks, Ian M. Rogers

**Affiliations:** 1 Lunenfeld Tanenbaum Research Institute, Sinai Health System, Toronto, ON M5G 1X5, Canada; aisha@lunenfeld.ca (A.M.); theresa.chow@mail.utoronto.ca (T.C.); jwhiteley@Lunenfeld.ca (J.W.); a.fantin@mail.utoronto.ca (A.F.); 2 Department of Physiology, University of Toronto, Toronto, ON M5S 1A8, Canada; kersti.sorra@mail.utoronto.ca; 3 BioPharmaceuticals R&D Cell Therapy Department, Research and Early Development, Cardiovascular, Renal and Metabolism (CVRM), BioPharmaceuticals R&D, AstraZeneca, 431 83 Mölndal, Gothenburg, Sweden; Ryan.Hicks@astrazeneca.com; 4 Soham & Shaila Ajmera Family Transplant Centre, University Health Network, Toronto, ON M5G 2C4, Canada; 5 Division of Reproductive Sciences, Department of Obstetrics and Gynecology University of Toronto, Toronto, ON M5G 1E2, Canada

**Keywords:** iPSC, umbilical cord tissue, mesenchymal stromal cells, episomal reprogramming, feeder free

## Abstract

The clinical application of induced pluripotent stem cells (iPSC) needs to balance the use of an autologous source that would be a perfect match for the patient against any safety or efficacy issues that might arise with using cells from an older patient or donor. Drs. Takahashi and Yamanaka and the Office of Cellular and Tissue-based Products (PMDA), Japan, have had concerns over the existence of accumulated DNA mutations in the cells of older donors and the possibility of long-term negative effects. To mitigate the risk, they have chosen to partner with the Umbilical Cord (UC) banks in Japan to source allogeneic-matched donor cells. Production of iPSCs from UC blood cells (UCB) has been successful; however, reprogramming blood cells requires cell enrichment with columns or flow cytometry and specialized growth media. These requirements add to the cost of production and increase the manipulation of the cells, which complicates the regulatory approval process. Alternatively, umbilical cord tissue mesenchymal stromal cells (CT-MSCs) have the same advantage as UCB cells of being a source of young donor cells. Crucially, CT-MSCs are easier and less expensive to harvest and grow compared to UCB cells. Here, we demonstrate that CT-MSCs can be easily isolated without expensive enzymatic treatment or columns and reprogramed well using episomal vectors, which allow for the removal of the reprogramming factors after a few passages. Together the data indicates that CT-MSCs are a viable source of donor cells for the production of clinical-grade, patient matched iPSCs.

## 1. Introduction

The advancements made over the past decade in cellular reprogramming have allowed for the first human clinical trials using autologous and allogeneic induced pluripotent stem cells (iPSCs) [1]. For clinical trial use, changes had to be made to the reprogramming protocols so that the iPSCs were generated under integration-free, feeder-free and viral-free conditions. A major roadblock with the first-generation reprogramming vectors was that they were of viral origin that required integration into the genome. Since then, non-integrating strategies, such as Sendai virus, episome and RNA, alleviate insertional risks such as mutagenesis, reactivation of silenced transgenes and tumorgenesis [2]. Episome reprogramming is currently being used in clinical trials in Japan [3].

The use of autologous iPSCs is being investigated for various diseases; however, many of these affect older patients such as in macular degeneration and cardiac disease [1,4]. This raises concerns with using cells from older donors for cell therapy since it has been determined that cells from older donors do not reprogram as efficiently as cells from younger donors and iPSC lines made from older donor cells have more DNA mutations [5]. With regard to cellular reprogramming, studies indicate that an apparent reversal of aging including telomerase reactivation, changes in methylation patterns and mitochondrial morphology, as well as a decrease in senescence markers such as p21, occurs [6,7]. However, despite these changes, cells from older donors reprogram less efficiently as compared to cells from younger donors [6,7]. Additionally, the resultant iPSC lines from older donors are known to have more DNA mutations [8]. Studies analyzing iPSCs generated from donors between the ages of 21 and 100 showed that mutations in the iPSCs increased linearly with age [9]. Several of the analyzed mutations were found to be linked to cancer or cell dysfunction. For instance, mutations in TET2, which is involved in somatic myelodysplastic syndromes, were observed [10]. Studies also demonstrated that demethylation during reprogramming is incomplete in older donors compared to younger donors, resulting in the retention of the epigenetic signature of the original somatic cell [9]. Lo Sardo et al. found that extended passaging of the iPSCs eventually erased most of the residual DNA methylation. However, the drawback to increased passaging is that prolonged culturing can also introduce aneuploidies and epigenetic variations. This, coupled with the increase in the amount of epigenetic and genetic abnormalities that occurs with age, can negatively affect the reprogramming efficiency of cells from older donors [9]. Of note, skin cells in particular are exposed to many environmental insults, such as UV radiation, that can damage the DNA over a human’s life span. Therefore, for future clinical applications, cells from younger donors are a promising cell source for reprogramming.

In search of a practical source of young cells, it has been argued that banked umbilical cord blood cells (UCB) can be a desirable donor cell type for generating iPSCs. Umbilical cord cells are the youngest cells that can be collected non-invasively and they can be stored for several decades without the loss of viability [11]. Furthermore, a source of very young cells is expected to not have accumulated many DNA mutations. Although genetic screening and analysis will still be required for iPSCs to be used in the clinic, there is a higher probability that a mutation-free cell line can be produced from younger versus older donor cells, thus saving time and money during iPSC production. Blood cells, whether from peripheral blood or the umbilical cord, require cell selection and cell-specific media to support the cells prior to reprogramming [12,13,14,15]. Antibodies for fluorescent activated cell sorting (FACS) or specialized cell isolation columns required for blood cell reprogramming would add hundreds of dollars and many hours of time to the process. Furthermore, the extra cell manipulation step can make the regulatory approval more difficult. The stromal cells within the umbilical cord therefore offer advantages over UCB cells.

Umbilical cord tissue mesenchymal stromal cells (CT-MSCs) can be isolated from explants of tissue without using collagenase, columns or FACS. The tissue pieces are banked without any cell selection and the isolation of mesenchymal stromal cells (MSCs) can occur after banking using the simple explant method. We have previously demonstrated using the explant method on n = 71 donors that MSC lines could be established from 100% of the donors [16]. Growing the explants in MSC-specific media ensured that only MSCs grew out of the explants and survived. Furthermore, many cells can be obtained after few passages since a single cord can yield millions of cells during the initial plating [16]. This is an advantage as reprogramming efficiencies have been shown to be negatively affected by the prolonged culturing of somatic cells before iPSC derivation. For example, the long-term culturing of fibroblasts has been shown to decrease reprogramming efficiency accompanied with an upregulation of p21, a marker indicative of cellular senescence [7]. Also, because collection and banking of CT-MSCs does not require an expensive collection bag or extensive preparation of the sample for storage compared to UCB, the cost of banking CT-MSCs is less. Thus, a large bank of donor CT-MSCs can be established at a lower cost than UCB banking [16,17]. Currently, there are millions of samples of UCB banked in public and private cell banks worldwide, while MSC banking is limited to private cell banks (https://parentsguidecordblood.org). However, with an increase in CT-MSC being used in clinical trials, public banking of CT-MSC will become a reality. Furthermore, UCB and CT-MSC banks can serve as a model for how iPSCs can be banked and distributed in the future [11].

The goal of this study was to use a clinically relevant reprogramming method to generate safe and cost-effective stem cells for therapy and drug discovery. Our project is the first to explore the feeder-free reprogramming of MSCs isolated from the umbilical cord tissue using episomal vectors. Being able to generate iPSC without the use of feeders will aid in achieving Good Manufacturing Practice (GMP) designation for the iPSC lines. A previous report on MSC reprogramming used feeder cells and determined that they did help with reprogramming efficiency [18]. Here, we chose to omit feeders and determine if MSC can still be reprogrammed efficiently. The CT-MSC were first characterized by surface marker identification and the ability to differentiate. Characterized CT-MSCs were then reprogrammed into iPSCs using the integration-free episomal vectors. Generated iPSCs lines were then characterized by morphology and for vector clearance, karyotype, expression of pluripotency markers and their differentiation capacity. Our findings support the possibility of utilizing umbilical cord tissue banks to generate human leukocyte antigen (HLA) typed iPSC lines (HLA-typed iPSC).

## 2. Materials and Methods

### 2.1. Umbilical Cord Tissue and Blood Collection and Preparation

Cord Tissue (n = 13) and umbilical cord blood (n = 2) were obtained from healthy full-term newborns from either vaginal or caesarean deliveries. Cord tissue was processed using a method developed in our lab as described in detail in Railenau et al. [16]. The cords were thoroughly decontaminated by wiping with 70% ethanol and then washed with phosphate-buffered saline (PBS) (−/−) to remove any residual blood. The cord was then sectioned into 5 mm thick sections. For cryopreservation, each of the 5 mm thick sections were divided into quarters and transferred to a 1.5 mL cryovial with a freezing solution made up of 80% MEM media (Gibco/Thermo, Mississauga, ON, Canada) with 10% Fetal Bovine Serum (Wisent, USA) + 10% dimethyl sulfoxide (Sigma Life Science, Missussauga, ON, Canada) + 1× Normocin (InvivoGen, USA). The tissue was then placed into a Mr. Frosty freezing container and stored at −80 °C. For appropriate cell preservation, the Mr. Frosty freezes the cells at approximately 1 °C/min. Samples were transferred to liquid nitrogen the next day for long-term storage. Sterility testing was conducted with BacT/ALERT Standard Aeorbic and BacT/ALERT Standard Anaerobic bottles (BioMerieux Inc., USA).

Umbilical cord blood cells were collected from healthy full-term neonates. The blood was processed and stored as previously described [19]. CD34+/Lin- cells were isolated using a lineage negative selection system from Stem Cell Technologies and the cells were grown for 8 days in Stemspan medium (Stem Cell Technologies, Canada): IMDM, 1% bovine serum albumin (*v*/*v*), 10 mg/mL bovine pancreatic insulin, 200 mg/mL human transferrin, 100 µM ß-mercaptoethanol, 2 mM L-glutamine supplemented with 40 mg/mL low-density lipoproteins (CalBiochem, San Diego, CA), 25 ng/mL FGF-4 (R&D Systems, MN, USA), 25 ng/mL SCF (R&D Systems, MN, USA), 25 ng/mL FLT3 ligand (R&D Systems, MN, USA) and heparin 25 ng/mL- referred to as FSFl-medium [19]. The expanded CD34+/Lin- cells were frozen and stored in liquid nitrogen prior to reprogramming.

### 2.2. Derivation of MSCs from Frozen Cord Tissue

Mesenchymal stromal cells (MSCs) were isolated using the non-enzymatic explant method. To recover the cryopreserved tissue, the cryovial was rapidly placed in a 37 °C water bath for 2–3 min. To remove any residual cryopreservant, the tissue pieces were immersed in 10 mL of complete MEM (α MEM (GIbco/BRL, Canada) with 5% Fetal Bovine Serum (Wisent, USA), 1× Normocin (InvivoGen, USA)). Single tissue pieces were plated in one well of a 6-well polystyrene dish (Falcon, Canada) and grown in complete MEM, allowing for the MSCs to migrate out onto the plate. The medium for these cord-tissue MSCs (CT-MSCs) was changed every 2 days. When cells reached 80% confluence (120,000 cells/cm^2^) they were passaged with a 0.25% trypsin–EDTA solution, neutralized with 10% serum, washed 1× and the contents of the plate were then transferred onto 10 cm plates (Falcon, Canada) and labeled as passage 1 (P1). Cells from passages 2–4 were frozen in multiple aliquots. These aliquots were then either used for characterizing the MSCs or for reprogramming.

For reprogramming, the CT-MSCs were grown in either serum-free medium (PRIME-XV MSC Expansion media, Irvine Scientific, USA) or complete MEM (Gibco, USA). For CT-MSCs grown using serum-free PRIME-XV MSC Expansion media (Irvine Scientific, USA), frozen aliquots were thawed and plated onto fibronectin-coated (Irvine Scientific, USA) 10 cm tissue culture plates at a density of 25,000 cells/cm^2^. Cells were kept in a 37 °C incubator at 5% CO_2_. A complete media change was done every 48 h and cells were passaged 1:4 at confluency onto 10 cm tissue culture plates. CT-MSC thawed using complete MEM were plated on tissue culture plates without any extra coating and cultured in the same way.

### 2.3. Flow Cytometry of Umbilical Cord MSCs Surface Markers

All CT-MSCs used for reprogramming were first characterized at early passage (P2-P4) using flow cytometry to confirm the expression of MSC cell surface markers. When the MSCs reached 80% confluency, the BD Stemflow hMSC analysis kit (BD Bioscience, Canada) was used to characterize the CT-MSCs. The cells were washed twice with PBS (−/−) and then harvested with Accutase (Sigma-Aldrich, Canada) for 5 min at 37 °C. The cells were then neutralized in 10 mL PRIME-XV MSC Expansion Media (Irvine Scientific, USA). The cells were centrifuged at 400× *g* for 4 min and resuspended in 1 mL stain buffer (PBS (−/−) + 1% FBS) at a concentration of 1 × 10^7^ cells/mL. The cells were then aliquoted into 9 tubes with each tube containing at least 1 × 10^6^ of cells for each antibody staining. Before the cells were stained with their respective antibodies, each tube was incubated with 2 µL of Fc block (anti-CD16/32 antibody) in the dark for 10 min to block any unspecific binding. Cells were stained separately with MSC markers: CD105 PerCP-CyTM5.5/ CD73 APC/ CD90 FITC (BD biosciences, USA) and the negative cocktail of hematopoietic markers: CD45/ CD34/ CD11b/ CD19/ HLA-DR PE (BD biosciences, USA) with the corresponding isotype control. Compensation controls were also done for each fluorophore to remove any spectral overlap. All the antibodies were stained with manufacturer’s recommendations. After the incubation, the cells were washed once with 1 mL of stain buffer and centrifuged at 400× *g* for 5 min at 4 °C. The cells were then quickly resuspended in 300 µL of stain buffer and 0.5 uL of 4′,6-diamidino-2-phenylindole (DAPI) (0.5 µg/mL). Samples were analyzed using the Gallios Flow Cytometer (Beckman Coulter, USA).

### 2.4. In Vitro Osteogenic Differentiation

Low-passage MSCs (P2-P4) were harvested from all donors and seeded at 5 × 10^3^ cells/cm^2^ in a 12-well plate in α-MEM/5% FBS. The next day the media was switched to the Osteogenic Differentiation Media (A1007201, StemPro, Gibco, Canada). The cells were fed every other day for 21 days. At the end of the 21 days, the media was removed from the wells and cells were rinsed once with PBS (−/−). The cells were then fixed with 10% formalin for 20 min at room temperature. After fixation, the cells were rinsed twice with distilled water and stained with 2% Alizarin Red solution (pH 4.2) for 3–5 min. The cells were finally rinsed four times with distilled water and kept in 400 µL PBS for subsequent imaging using a light microscope.

### 2.5. In Vitro Adipogenic Differentiation

Low-passage MSCs (P2–P4) were seeded at 5 × 10^3^ cells/cm^2^ in a 12-well plate in complete MEM. The media was switched the next day to Adipogenic Differentiation Media (#A1007001 StemPro, Gibco, Mississauga, Canada) and the cells were then grown for 14 days while being fed every other day. At the end of the 14 days, the media was removed from the wells and the cells were washed with PBS (−/−), fixed with 10% formalin for 20 min and rinsed with PBS (−/−). Before staining, the cells were washed with 60% isopropanol for 5 min then stained with a freshly made working solution of Oil Red O for 15 min. The cells were washed four times and kept in 400 µL PBS for subsequent imaging.

### 2.6. Episomal Reprogramming

For reprogramming, the CT-MSCs were grown in PRIME-XV MSC Expansion Media (Irvine Scientific, USA) or complete MEM (Gibco, Canada). Human iPSCs were generated using viral-free and non-integrative episomal vectors (Epi5) (Invitrogen, Canada), which carried the reprogramming factors Oct4, Sox2, Lin28, Kld4 and L-myc along with a p53 short-hairpin RNA. One day, prior to transfection, the CT-MSCs were cultured in antibiotic-free media until they were 75–90% confluent. The cells were then detached using Accutase (Stem Cell Technologies, Canada). Approximately 1 × 10^5^ to 1 × 10^6^ cells were transfected with 1 µL of each vector from the Epi5 kit (Invitrogen, USA) using the Neon transfection system (Invitrogen, cat #MPK5000) with the parameters, 1150mV-30ms-2 pulses and 1650mV-30ms-2 pulses. The electroporated cells were then plated onto Geltrex-coated 10 cm plates or 6-well plates (Thermo Fisher, Canada) at 18,000 cells/cm^2^) when complete MEM was used to a low density (1800 cells/cm^2^) with PRIME-XV MSC Expansion Media (Irvine Scientific, USA) and incubated for 24 h. On Day 2, the cells were switched to N2B27 Media (DMEM/F12 with 25 mM HEPES (Invitrogen, USA) and 1X N2 supplement (Invitrogen, USA), 1× B27 supplement (Invitrogen, USA), 10 mM MEM NEAA (Invitrogen, USA), 1× Glutamax (Invitrogen, USA), 55 µM B-mercaptoethoanol (Invitrogen, USA), and 100 ng/mL bFGF, 50 µg/mL of ascorbic acid (Stem Cell Technologies, Canada). The N2B27 media was changed every other day for 7 days and then every day for the remaining 2 days. On Day 9, the N2B27 media was replaced with NutriStem media (Biological Industries, USA). This media was changed every other day. Colony formation was noted from Day 21–26, after which colonies were picked for expansion.

### 2.7. Verification of Episome Vector Removal

iPSCs were grown in Nutristem media on Geltrex-coated plates and passaged with Accutase (Stem Cell Technologies, Canada). For rapid vector clearance, the iPSCs were switched to mTeSR1 medium (Stem Cell Technologies, Canada) on Vitronectin-coated plates (Stem Cell Technologies, Canada) and passaged with ReLeSR (Stem Cell Technologies, Canada). After vector clearance, iPSCs could be passaged successfully using either cell culture system (Nutristem media with Geltrex-coated plates or mTeSR1 media with Vitronectin-coated plates). DNA was isolated from iPSCs by lysing the cells in 100 µL of 0.05 M NaOH and incubating for 10 min at 98 °C. The reaction was then neutralized using 10 µL of 1 M Tris (pH 8.0) and the DNA was further diluted by adding 100 µl of molecular-grade sterile water. The following primers were used: oriP (Forward Primer: 5′-TTC CAC GAG GGT AGT GAA CC-3′, Reverse Primer: 5′-TCG GGG GTG TTA GAG ACA AC-3′) and EBNA-1 (Forward primer: 5′-ATC GTC AAA GCT GCA CAC AG-3′, and Reverse Primer: 5′-CCC AGG AGT CCC AGT CA-3′) for detection of the Epi5 vectors, and GAPDH (Forward primer: 5′CGAGATCCCTCCAAAATCAA3′, Reverse Primer: 5′GTCTTCTGGGTGGCAGTGAT3′) as a house-keeping gene. All primers were diluted to a final concentration of 5 µM. PCR amplification was done with a total volume of 12.5 µL with 100 ng of genomic DNA. PCR samples were then run on a 1.5% agarose gel with 1× Sybrsafe dye and 100 bp ladder (Froggo, USA). All of the gels were visualized using UV fluorescence.

For the UCB iPSCs, colonies were picked, passaged and subcloned.

### 2.8. Immunocytochemistry

The iPSCs were seeded onto 12-well plates coated with Geltrex, grown to confluency, washed twice with PBS (−/−) and fixed with 10% formalin for 20 min at room temperature. The fixed cells were washed with PBS (−/−) then incubated with blocking buffer (10% FBS, 0.1% in PBS) for 30 min at room temperature to prevent any non-specific antibody binding. The primary antibodies were prepared by diluting with antibody buffer (0.2% FBS, 0.1% Triton X-100 in PBS stored at 4 °C). The following primary antibodies were used: Oct4 (1:100 dilution, Invitrogen), Sox2 (1:100 dilution, R&D Systems, USA) and Nanog (1:10 dilution, ReproCell, USA). After blocking, the wells were washed once with PBS (−/−) with 0.1% Triton X-100 and 300 µl of the primary antibody was added to each well. Primary antibodies were incubated overnight at 4 °C. After primary antibody incubation, the cells were washed (4 × 15 min) and the respective secondary antibodies were added to the cells and then incubated for 30 min at room temperature in the dark. Afterwards, the cells were washed (5 × 15 min) and stained with DAPI. All fluorescence images were taken on the spinning disk confocal (Quorum WaveFX Spinning Disc Confocal System). Secondary antibody controls were done to show no cross-reaction with the tissue (Figure S1).

### 2.9. Karyotyping

The iPSCs lines were sent to the Cytogenomic Services at the Hospital for Sick Children (Toronto, Canada) for karyotyping. Karyotype analysis via G-banding was performed on cells from two 6-cm dishes per cell line. Routine G-banding analysis was then carried out. Twenty metaphases per cell line were examined.

### 2.10. Embryoid Body Formation

The iPSCs were cultured in mTeSR on Vitronectin-coated 6-well plates. Once 60–70% confluent, the cells were dissociated using ReLeSR (Stem Cell Technologies, Canada). To form embryoid bodies (EBs), the harvested iPSCs were set up as hanging drops at 25,000 cells/25 µL drop in DMEM/F12 + 20% knockout serum replacement (KSR, Invitrogen, USA), 2 mM L-glutamine, 100 µM nonessential amino acids, 100 µM 2-mercapto-ethanol (Invitrogen, Canada), 0.5% penicillin and streptomycin, and 10 µm ROCK inhibitor (Y-27632:Tocris, Canada). The hanging drops were left for 72 h at 37 °C to allow for self-aggregation. On Day 3, the EBs were flooded with 10 mL DMEM + 10% FBS and the media was changed every other day for 4 days. The aggregates were then transferred to 12-well polystyrene tissue culture plates, where the EBs began to flatten. The media was changed every 48 h for two weeks. On Day 14, immunocytochemistry was conducted and EBs were stained with primary antibodies to ßIII-Tubulin (1:100 dilution, Millipore, USA) to detect ectoderm, Smooth Muscle Actin (1:100 dilution, Dako, USA) to detect mesoderm and HNF3ß (1:100 dilution, Santa Cruz, USA) to detect endoderm.

### 2.11. Teratoma Formation

All mouse injections were carried out at The Centre for Phenogenomics (TCP; Toronto, Canada) in accordance with the guidelines of the Animal Care Committee. To form teratomas, 1 × 10^7^ cells were mixed with high-concentration Matrigel (BD Bioscience, USA) and DMEM at a 1:1 ratio and injected subcutaneously into the hind limb (one injection per side) of NOD/SCID (NOD.Cg-Prkdcscid/J) mice (JAX, USA cat#001303). The mice were monitored for tumors over the course of 12–15 weeks and euthanized with CO_2_ once tumor growth was palpable and visibly noticeable. Tumors were fixed in 10% NBF overnight, embedded, and stained for hematoxylin and eosin (H&E). Histology of the teratomas was assessed by the staff pathologist.

### 2.12. Statistical Analysis

Data are presented as the mean standard error (SE) of five independent experiments. Statistical significance was determined by an unpaired, two-tailed Student’s *t*-test. * *p* < 0.05. A correlation coefficient was analyzed by the Pearson test.

## 3. Results

### 3.1. Establishment and Characterization of CT-MSC

**Figure 1:**
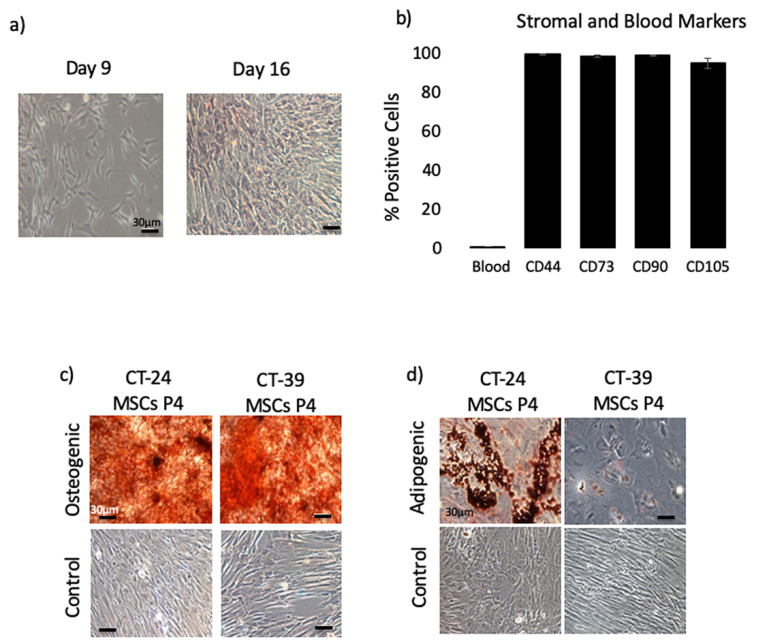
Characteristics of umbilical cord tissue mesenchymal stromal cells (CT-MSCs) isolated through the explant method from cryopreserved primary human umbilical cord tissue. (**a**) Cell outgrowth at Day 9 and Day 16 post-plating. The cells have a normal spindle-shaped morphology and became confluent after 16 days. (CT-MSC 200). Scale bar =30 µM for all pictures. (**b**) The cell lines are over 95% positive for the MSC markers (CD44, CD73, CD90 and CD105) and < 1% positive for the hematopoietic markers (‘blood’- pooled; CD34, CD11b, CD19, CD45 and HLA-DR) at passage 4 (n = 5 cords). Standard Error bars (red) are shown. No statistical difference between passages was observed. (**c**) Passage 2-4 CT-MSCs were cultured in osteogenic differentiation medium for 14 days and stained with 2% Alizarin Red solution for calcium deposits. The control well was grown in α MEM+5% FBS and showed no positive staining. Scale bar = 30 µM for all panels. (**d**) Passage 2-4 CT-MSCs were cultured in adipogenic differentiation medium for 14 days and stained with Oil Red O to stain lipid droplets. The control well was grown in α MEM+5% FBS and showed no positive staining. Scale bar = 30 µM for all panels.

Before reprogramming, the CT-MSCs were characterized according to an internationally recognized set of criteria including fibroblast morphology, adherence to plastic and expression of MSC markers [20]. Although multi-lineage differentiation is no longer a criterion, as it has been demonstrated that MSCs are a multipotent population not multipotent cells, we included adipocyte and osteoblast differentiation in our analysis. CT-MSCs were isolated from tissue explants (n = 13) and the morphology of the cells were routinely monitored. Cells with adherent fibroblast morphology began to grow out of the tissue pieces between 5–15 days depending on the donor sample. Cells grew out of all the tissues tested as a uniform parallel sheet of spindle-shaped cells (Figure 1a). After the first passage the cells were subsequently expanded for one additional passage then cryopreserved for further characterization. Thawed cells (P2-P4) were grown in PRIME-XV MSC Expansion media and were characterized using flow cytometry to measure the expression of MSC cell surface markers. All the cells stained positive for MSC markers and negative for hematopoietic markers. The CT-MSCs from 13 donors expressed CD44 (99.5% ± 0.08569), CD73 (94.108% ± 0.5229), CD90 (99.23% ± 0.2648) and CD105 (95.08% ± 2.333). Less than 1% of the cells were positive for the blood markers (CD34, CD11b, CD19, CD45 and HLA-DR) (Figure 1b and Figure S2). Additionally, P2-4 MSCS were found negative by ICC for CD31, a marker for endothelial cells. The media used to grow CT-MSC is not supportive of endothelial cells and any that might be present during the initial isolation do not survive (Figure S3).

To assess multi-lineage differentiation, the CT-MSCs were differentiated into bone (osteoblasts) and fat (adipocytes). Differentiated osteocytes displayed extensive staining for Alizarin Red, which stains for calcium deposits (Figure 1c). Differentiated adipocytes displayed positive staining for accumulated lipid droplets in the cytoplasm, which are stained with Oil Red O (Figure 1d). Undifferentiated CT-MSCs grown in complete MEM were used as control cells and they stained negative for both Oil Red O and Alizarin Red (Figure 1c,d).

### 3.2. iPSC Reprogramming

The Epi5 reprogramming protocol (Invitrogen) uses a mixture of five vectors that contain five reprogramming factors (OCT4, SOX2, LIN28, KLF4, and L-MYC) to generate transgene-free, virus-free iPSC lines. Optimization experiments with the Epi5 vectors demonstrated that 1150 mV-30 ms-2 pulses and 1650 mV-30 ms-2 pulses gave the most colonies.

We also tested different seeding densities as the high proliferation rate of the cells during reprogramming could mask initial colonies if the cells were slow to reprogram. Conversely, too low a cell density at the start of reprogramming would reduce cell–cell communication that is important for iPSC colony initiation [21]. We previously observed very little variation in the rate of cell proliferation between different MSC donor samples (n = 71) [16]. We did notice that the MSCs grew very well in the PRIME-XV MSC Expansion medium reaching confluence more rapidly than what was observed with α MEM + 5% FBS. During the 4 days between passages, the MSCs in the PRIME-XV MSC Expansion medium had 1–2 extra cell divisions resulting in 2–4× more cells compared to the complete MEM (Figure S4). Post-electroporation plating densities ranged from a high density (18,000 cells/cm^2^) when complete MEM was used to a low density (1800 cells/cm^2^) when PRIME-XV MSC Expansion medium was used. The final electroporation parameters used were 1650 mV-30 ms-2 pulses for cells in the PRIME-XV MSC Expansion and 1150 mV-30 ms-2 pulses for cells grown in complete MEM. Of note, the higher voltage resulted in increased frequency of transfected cells but also higher cell death. The Epi5 vector was used at 1µL/10^5^ to 10^6^ cells. Despite the recommendation of 1µl/10^5^ cells we achieved colonies at the higher cell number. We also found that the choice of media did not alter the efficiency of reprogramming as CT39 was tested in both media with similar results.

**Figure 2:**
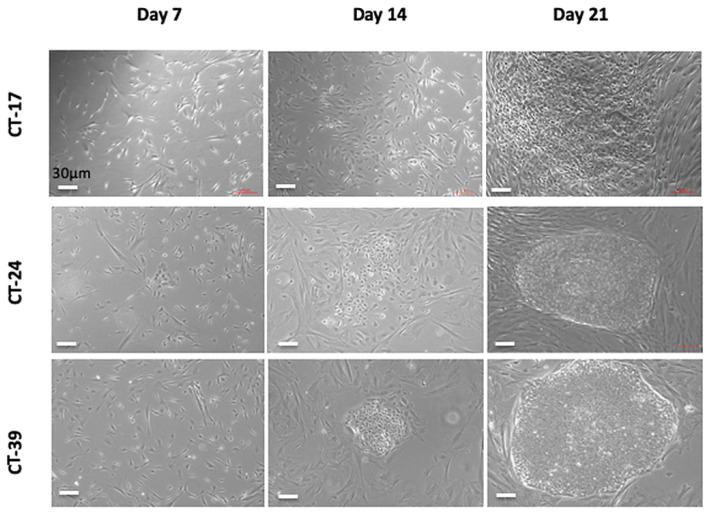
Observed changes to the CT-MSC during reprogramming. The CT-MSCs’ transition throughout the 21 days of reprogramming. Colonies present with a human embryonic stem cell-like morphology with defined boarders and a high ratio of nucleus to cytoplasm. The colonies were then picked and expanded for further characterization. Scale bar = 30 µM for all panels.

During the process of reprogramming, the cells undergo specific morphological changes. At the initiation of reprogramming, the cells undergo a mesenchymal to epithelial transition (MET). This transition can be seen as cells shift from a fibroblast-like morphology into loosely packed clusters of rounded cells. By Day 14, areas of reprogrammed cells were observed to be emerging (Figure 2). By Day 21, the cells showed visible compact colonies with defined boarders like a typical embryonic stem cell (ESC) colony (Figure 2). Colonies were picked, expanded and frozen down for characterization.

iPSC lines were obtained from 12 of the thirteen CT-MSC donors for a 92% reprogramming success rate (Table 1). All CT donor samples produced healthy MSCs that grew well over multiple passages in either serum-free (PRIME-XV MSC Expansion) or serum-containing (complete MEM) medium. However, not all donor cells were able to complete reprogramming (Table 1). When the PRIME-XV MSC Expansion medium was used, seven out of 10 CT-MSC donors produced sustainable colonies. When complete MEM medium was used, six out of seven donor samples were able to reprogram. CT56 failed to reprogram under either condition despite multiple attempts. CT56 produced three colonies but they failed after four passages. The reason for this donor’s failure to produce sustainable iPSC colonies is unknown. CT 39 reprogrammed well in both media.

To determine if a modifier would increase the reprogramming efficiency of CT-MSC reprogramming, ascorbic acid, a natural compound that has been shown to enhance iPSC generation, was tested on a subset of CT-MSC donor cells. Other studies have shown that the addition of ascorbic acid can boost reprogramming [22]. As a control, two parallel experiments were conducted, one with 50 µg/mL ascorbic acid and one without ascorbic acid. Of the five donors tested, all reprogrammed without ascorbic acid supplement and no enhancement was observed with ascorbic acid supplementation. The MSC line that did not produce any colonies during the initial attempt, did not produce colonies with the addition of ascorbic acid. Despite the enhancement in reprogramming using ascorbic acid observed by others, ascorbic acid for CT-MSC reprogramming was not beneficial in the conditions of our experiment.

### 3.3. Confirming Pluripotency of the CT-MSC Derived iPSC

The pluripotency of cells is defined by their ability to produce tissue from all three germ layers: ectoderm, mesoderm, endoderm [23]. To confirm the pluripotency of the CT-MSC derived iPSCs, both in vitro differentiation through embryoid body formation and in vivo differentiation through teratoma formation were used. While morphology and gene expression have also been used to examine iPSCs, our previous studies have demonstrated ‘alternative’ states of reprogramming where pluripotency is retained although morphology and gene expression present differently than expected [24]. Additionally, the use of different coatings for culture plates or feeder cells can also alter the morphology of iPSC colonies. Before pluripotency can be assessed, however, the confirmation that the episomal vectors have been cleared from the iPSC lines is required.

**Figure 3:**
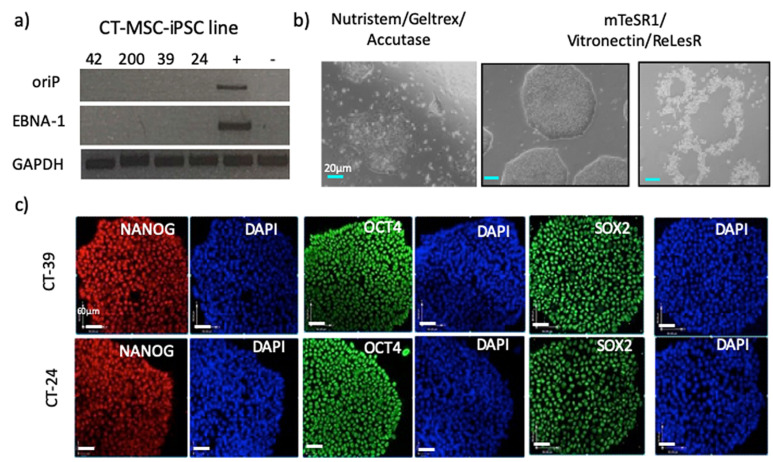
Vector-free pluripotency cell lines established from CT-MSC. (**a**) The CT-induced pluripotent stem cell (iPSC) lines are negative for the OriP and EBNA genes of the episomes carrying the reprogramming factors by passage 10, indicating the loss of the episome after reprogramming has completed. The positive control (+) is an earlier passage 3 of the CT-42 iPSCs lines still containing the reprogramming vectors, and the negative control (−) is a human embryonic stem cell (ESC) line (CA1). (**b**) Phase contrast images of the morphology of CT-MSC iPSCs grown in Nutristem on Geltrex matrix compared to mTeSR on Vitronectin matrix. Distinct, uniform colonies with defined borders are observed only with mTeSR1 medium with Vitronectin-coated plates. The iPSC colony can be easily lifted from the plate using ReLeSR leaving behind the differentiated cells that sometimes form at the outer edges of pluripotent stem cell colonies. Scale bar = 20 µm for all panels. (**c**) Vector-free CT-MSC-iPSC lines express pluripotency markers Nanog, Oct4 and Sox2 as detected by immunocytochemistry. Scale bar = 60 µm for all panels.

Episomal plasmids replicate in the nucleus of the cell extra-chromosomally but at a slower rate than the nuclear DNA and therefore can be diluted out and eventually removed from the cells after several cell divisions. End-point PCR using primers specific to the episome vector genes EBNA-1 and oriP, were used to confirm whether the established iPSCs were free of all the transgenes. To check for the presence of reprogramming vectors, DNA was collected from the iPSCs from passage 4 and above. Different media and substrate combinations were tested to determine if vector clearance was different depending on the growth conditions of the nascent iPSC. In the first set of conditions, the cells were grown in the Nutristem media and Geltrex-coated plates and passaged with Accutase. Under these conditions, cells retained the reprogramming factors up to passage 10. The colonies were fairly defined but not completely uniform. We hypothesized that the difference in cell morphology indicated a mixture of vector-free and vector-containing cells. When early passage iPSCs were instead cultured in mTeSR1 media on Vitronectin-coated plates, vector-free lines could be generated between passage 4–10, as seen by negative PCR for the episomal vector (Figure 3a). Additionally, after switching to the dissociating agent ReLeSR instead of Accutase, the most pluripotent cells were lifted and passaged while the differentiated cells along the outer edges were left behind (Figure 3b). These iPSCs were grown for multiple passages using mTeSR1 media, Vitronectin coating and ReLeSR then tested for the expression of pluripotency proteins Oct4, Sox2 and Nanog by ICC (Figure 3c). All CT-MSC derived iPSC lines expressed all three pluripotency proteins, and a comparison to DAPI indicates that all of the cells were positive for Oct4, Sox2 and Nanog. Having confirmed that the lines were episome free, further in vitro and in vivo characterization was conducted.

**Figure 4:**
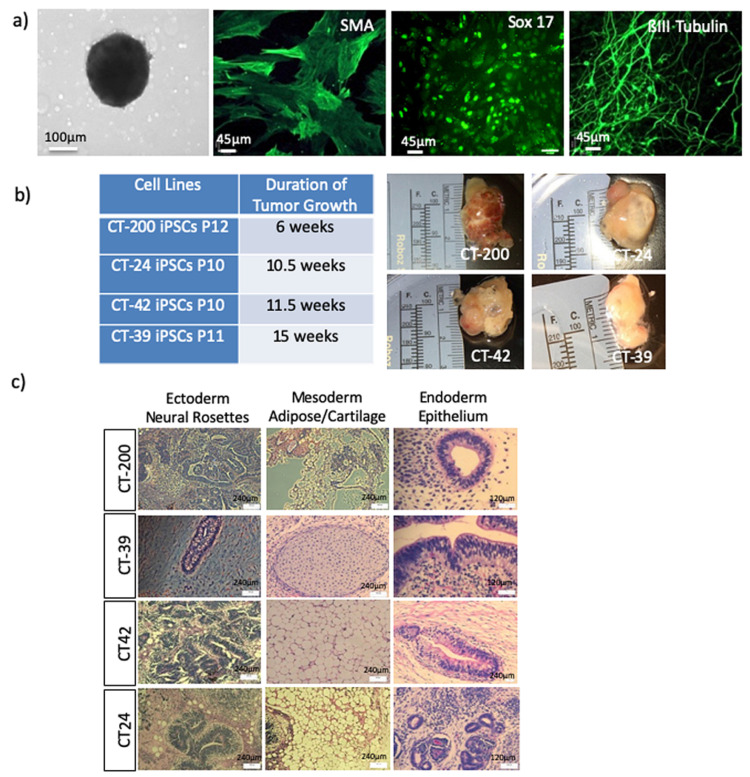
Embryoid body mediated multi-lineage differentiation of CT-MSC iPSCs. (**a**) Phase contrast images show the embryoid bodies (EBs) formed by each of the iPSCs lines (Scale bar = 100 μm) and immunofluorescence shows the differentiation of the iPSCs into the three germ layers ectoderm (ßIII-Tubulin), mesoderm (smooth muscle actin—SMA) and endoderm (Sox17) in vitro. Scale bar = 45 μm. (**b**) Reprogrammed transgene-free iPSCs successfully generated subcutaneous teratomas with similar morphology. Representative images of teratomas generated after injecting 1 × 10^7^ iPSCs into the hind limb of NOD/SCID mice. Teratomas were excised between 6–15 weeks after the subcutaneous injection of cells. (**c**) The isolated tissue was processed and stained with hematoxylin and eosin (H&E) to identify the three germ layers with neural rosettes (ectoderm), epithelium structures (mesoderm) and adipose and cartilage tissue (endoderm).

The embryoid body (EB) assay is an in vitro assay used to determine the ability of CT-MSC derived iPSCs to differentiate into the three germ layers (ectoderm, mesoderm and endoderm). The iPSC will aggregate in the hanging drops and begin to form a gastrulae that will spontaneously differentiate into ecoderm, mesoderm and endoderm [25]. The iPSC lines successfully generated EBs when grown in hanging drop culture. The EBs were relatively similar in size and shape for all of the lines. After 3 days in hanging drops, the EBs were covered with media (DMEM+10% FBS) and placed onto adherent tissue culture plates to stimulate spontaneous differentiation. After growing the EBs for 2 weeks post-adherence, immunocytochemistry was performed for the presence of ectoderm (ßIII-Tubulin), mesoderm (Smooth Muscle Actin) and endoderm (HNF3 ß) markers. Immunostaining of the EBs shows that the cells were positive for the ectodermal marker ßIII-Tubulin, the mesodermal marker alpha smooth muscle actin (α SMA) and endodermal marker HNF3ß (Figure 4a).

The teratoma assay was carried out to assess in vivo differentiation of the iPSCs lines through the subcutaneous injection of 1 × 10^7^ cells into the dorsal flanks of NOD/SICD mice. The time for palpable tumors to form varied between iPSC lines. Visible growth was seen in the mice anywhere between 4–15 weeks after injection (Figure 4b). All injected lines successfully generated tumors representative of all three germ layers: neural tube (ectoderm), adipose and cartilage (mesoderm) and gut epithelium (endoderm) as assessed by the staff pathologist (Figure 4c).

**Figure 5:**
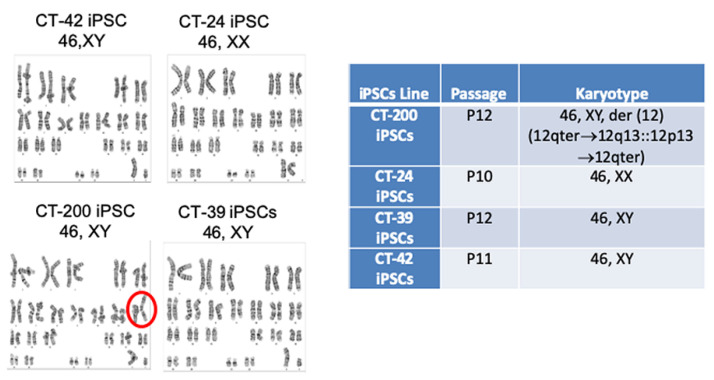
Karyotype analysis of iPSC derived from CT-MSC. Karyotype detected by G-band analysis of representative iPSC lines demonstrates that CT-42 iPSCs and CT-39 iPSCs carry a normal diploid male karyotype and CT-24 iPSCs carries a normal diploid female karyotype. CT-200 iPSCs carries a mostly normal diploid female karyotype with an abnormal chromosome 12 (red circle).

To assess the genomic stability of the iPSCs, karyotyping was performed. For karyotype analysis, the cells must be dividing and be at the late metaphase stage. The resulting G-band was analyzed for any large structural chromosomal aberrations. The testing of 20 metaphase cells is the universal standard by regulators and any abnormalities found in two separate samples would conclude that the line is abnormal. Passage 12 CT-200 iPSCs showed an abnormal karyotype. In total, 100% of the metaphase cells analyzed showed 46 chromosomes with a trisomy in chromosome 12. This was due to an additional chromosome segment added to the distal band 12q13 attached to the short arm of chromosome 12 (Figure 5).

**Figure 6:**
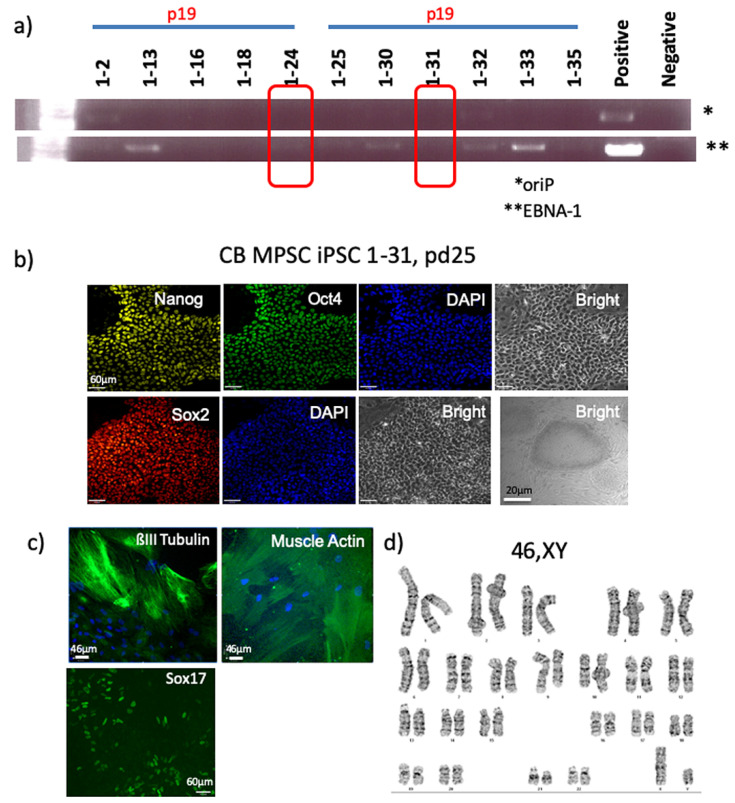
Umbilical Cord Blood Reprogramming. (**a**) PCR demonstrating the loss of episome vectors from reprogrammed umbilical cord blood (UCB) cells. The episome vector is not present in UCB-iPSC lines 24 and 31. (**b**) Episome-free UCB-iPSC are positive for the pluripotency markers Oct4, Sox2 and Nanog. Scale bar = 60µm for all ICC panels and scale bar = 20µm for the phase contrast panel. (**c**) UCB-iPSC were successfully differentiated into ectoderm (ßIII-Tubulin), mesoderm (SMA) and endoderm (Sox17). Scale bar = 46µm for all panels. (**d**) A normal diploid male karyotype is observed.

As a control for the Epi5 reprogramming system we used umbilical cord blood (UCB) cells. One million UCB cells were transfected using the NEON electroporation system and subsequently plated onto MEFs, resulting in 128 colonies at Day 21 of reprograming. In a second experiment, 3.2 million cells were transfected and cells were plated onto geltrex treated plates (no MEFs), resulting in 24 colonies at Day 21 of reprogramming. A total of 12-24 clones were randomly chosen to establish stable lines from both experiments (Figure 6). The control UCB Lin-/CD34+ iPSC colonies were picked, passaged and tested for vector clearance. An example of two episomal free lines is shown (Figure 6a). The episome-free lines were then tested for pluripotency proteins. The lines were positive for Oct4, Sox2 and Nanog (Figure 6b). Tri-lineage differentiation through spontaneous differentiation revealed that the iPSCs could produce mesoderm (α SMA), endoderm (ßIII-Tubulin) and endoderm (Sox17) (Figure 6c). Chromosome analysis revealed a normal 46XY Karyotype (Figure 6d).

## 4. Discussion

Since the discovery of iPSCs in 2006, the field of regenerative medicine has changed drastically. In order for iPSCs to be used clinically, the optimization of the starting donor cell type and the reprogramming protocol must meet certain criteria. Critical criteria are that the donor cells should be mutation free, easily obtained or harvested and amenable to reprogramming. The reprogramming protocol should result in a high efficiency of reprogramming and produce transgene-free iPSC lines. There are now a wide range of methods available to generate iPSCs [2]. The episomal-based vector system, Epi5, is being used to generate iPSCs for clinical use as it allows for transgene-free iPSC production [26].

Numerous factors can affect reprogramming, including the chosen reprogramming protocol, culture media, cell type as well as the cell age and passage number. Ideally, an autologous cell source would be used. However, many of the diseases that will be treated using iPSCs are diseases associated with advanced age. Autologous cells, therefore, may not be the optimal source due to the implications that aged cells retain their DNA mutations during reprogramming [9]. Cells sourced from young donors, such as umbilical cord blood or umbilical cord tissue cells are an attractive alternative. Private and public umbilical cord banks are well established and contain donor samples of umbilical cord blood and tissue cells [27]. Furthermore, the banked cells are currently stored in a clinically complaint manner. As such, these facilities have the capability to support the manufacturing of clinical-grade iPSCs. Although UCB has been well documented as a source of donor cells for reprogramming, there are few publications demonstrating the successful reprogramming of MSCs using a clinically relevant reprogramming system. Here, we chose to use the episomal system (Epi5) to investigate the reprogramming of CT-MSCs because episomal vectors are currently being used in clinical trials [1].

The CT-MSCs isolated from umbilical cord tissue have several advantages as a donor cell population. They can be cryopreserved and stored for many years without the loss of viability and they are collected non-invasively. We previously demonstrated on a large set of CT-MSC donors (n = 71) that stable MSC lines could be established from 100% of frozen, banked donor cords. This removes a significant variable seen with other donor cell sources [6]. Furthermore, umbilical cord tissue banks provide a variety of HLA diverse donors that could be used for the establishment of a histo-compatible iPSC supply. Currently, public umbilical cord blood banks do not bank cord tissue MSCs, but as the number of clinical trials using CT-MSC increases, the public banking of CT-MSC should commence [28].

iPSC lines have already been successfully established using umbilical cord blood, but, for all of the reprogramming protocols in use, cell selection must be done prior to reprogramming [26,29,30,31]. The cell selection step uses columns and additives that add an extra cell manipulation step that has to be considered during regulatory approval. Using CT-MSCs eliminates the extra step of cell selection using antibodies and/or columns making regulatory approval easier. We hypothesized that CT-MSCs would offer the same age, collection and banking advantages of umbilical cord blood with the added benefit of a simple, minimally manipulated preparation of cells for reprogramming.

To circumvent any lot-to-lot variability, animal pathogen concerns and scalability challenges, it is important to use a defined feeder-free reprogramming and cell culture system [32]. For the derivation of the CT-MSC-iPSC lines, a chemically defined serum-free MSC media is beneficial. Here, we demonstrated that the CT-MSCs grew very rapidly in the Prime-XV MSC Expansion serum-free media as the starting cell numbers and plating density had to be adjusted for the increased proliferation rate compared to the standard culture media of 5%FBS/α-MEM. The ability to obtain a large number of cell outgrowths from the cord tissue pieces combined with the robust proliferation rate provided a sufficient number of cells within 2-6 doublings, which is advantageous as low-passage cells have higher rates of reprogramming [7]. Despite the advantage of using serum-free medium during CT-MSC growth compared to the serum-containing media, the reprogramming efficiencies were similar in both medium.

Since UCB cells have been used to successfully generate iPSC lines with episome vectors, we chose to use UCB as a positive control for our experiments [26]. The UCB control yielded a similar number of colonies per 100,000 cells as observed for CT-MSC donors when feeder-free, geltrex treated plates were used post-transfection. Although only one experiment was done using a feeder co-culture, we observed that the yield of colonies was higher. Despite the increase in iPSC colonies with feeders, the use of feeders is not amenable to developing clinical-grade iPSC lines.

Only one donor CT-MSC sample (CT56) failed to produce a stable iPSC line even though the CT-MSC grew well in serum-free medium prior to transfection. CT56 did produce three iPSC colonies that could be isolated and passaged but did not remain stable and reverted back to MSC-like cells. During the multiple attempts to generate a stable iPSC line from CT56, feeders were introduced into the culture to determine if they could provide the missing factor that was preventing full reprogramming. However, the addition of feeders did not solve the problem. Neither did the addition of ascorbic acid. Since all donor samples are received blinded and anonymously, we were unable to obtain more information on the donor or the birth circumstances to determine why this one donor failed to reprogram.

Karyotype analysis is necessary for the complete characterization of iPSC lines. Additionally, a major concern of genetic and epigenetic variations in iPSCs is the possible negative effect when used for cell-based therapies and disease modeling. It is important to assess the genetic stability of the cells as there can be serious implications if mutated, undifferentiated, or incorrectly differentiated cells are transplanted. Epigenetic and genetic aberrations can be acquired through reprogramming and prolonged culturing of cells [33,34]. The CT-200 iPSC line was tested at passage 12 and all 20 metaphase cells carried a partial trisomy 12 mutation, with duplication in the segment distal to the band 12q13 attached to the short arm of chromosome 12. It is still unknown how these mutations are acquired. It has been previously suggested that during prolonged culture conditions mutations from the reprogramming process and mutations carried over from the somatic cells can cause an increase in genetic instability [35]. Maintenance of a stable karyotype is crucial for clinical cell-based therapies and reproducibility in basic science research. It is common that karyotype abnormalities can cause some cells to gain a selective advantage in culture. Partial and full trisomy of 12 chromosomes is one of the most common abnormalities in iPSCs. Nanog, a protein critical for pluripotency, resides on the short arm of chromosome 12 and likely offers a growth advantage to colonies containing an extra copy of Nanog [35]. Other common aberrations in human iPSC lines include trisomy in chromosome 8 and chromosome X [36]. These common mutations are usually detected in a subpopulation of the cells, and, with passaging, these cells seem to survive and eventually take over the culture. This suggests that cells bearing these abnormal karyotypes have a selective advantage that allows them to grow and proliferate very robustly.

## 5. Conclusions

In this study, stable CT-MSC lines could be successfully isolated and cryopreserved from human umbilical cord tissue MSC donors. A viral-free and integration-free protocol to reprogram CT-MSCs into iPSCs using the episomal method in feeder-free conditions was established. The iPSC lines demonstrated positive staining for pluripotency markers Oct4, Sox2, and Nanog. The iPSC lines successfully differentiated into all three germ layers as tested with embryoid body culture and the teratoma assay. Finally, three out of the four iPSCs that were karyotyped were genetically stable after 10 passages. We have demonstrated here that CT-MSCs are a potential source of young cells that can be used to generate iPSC lines. Private and public umbilical cord tissue banks could provide the starting cells needed to establish iPSCs lines for transplantation for HLA-matched patients.

## Figures and Tables

**Table 1:** Culture conditions used to grow the CT-MSCs prior to reprogramming and reprogramming result.

CT#	Numbers of Colonies Observed	
	Serum Free	+FBS
17	1	not tested
24	7	not tested
26	1	not tested
42	7	not tested
200	8	not tested
201	7	not tested
39	11	39
16	0	2
21	0	16
56	0	0
28	not tested	5
46	not tested	6
15	not tested	7

Both serum-free and serum-containing medium was used to culture the CT-MSCs prior to reprogramming. A total of 70% of the serum-free CT-MSCs reprogrammed, while 85% of the serum-containing CT-MSC reprogrammed. One sample did not reprogram when grown in either media (CT-56) and one sample reprogramed well when grown in either media (CT-39).

## Data Availability

All data are included in the paper. There are no databases associated with this manuscript.

## Supplementary Materials

The following are available online at https://www.mdpi.com/2073-4409/10/1/49/s1, Figure S1: Secondary Antibody Controls; Figure S2: Flow cytometry dot plots for five of the CT-MSC lines used for reprogramming; Figure S3: CT-MSC are negative for endothelial cells; Figure S4: CT-MSC proliferate more in serum free media versus serum containing media.

## Abbreviations

CT-MSC: Cord Tissue Mesenchymal Stromal Cells. UCB: Umbilical Cord Blood. iPSC: induced pluripotent stem cells. MEF: Mouse embryonic fibroblasts. EB: Embryoid Body. HLA: Human Leukocyte Antigen.
