# Supplementary material for: Umbilical Cord Tissue as a Source of Young Cells for the Derivation of Induced Pluripotent Stem Cells Using Non-Integrating Episomal Vectors and Feeder-Free Conditions
Source: Cells. 2020 Dec 31;10(1):49. doi: 10.3390/cells10010049 (PMC7824218; doi:10.3390/cells10010049)
Supplement: Supplementary file 1 [file cells-10-00049-s001.pdf]

Article

Umbilical Cord Tissue as a Source of Young Cells for the Derivation of Induced Pluripotent Stem Cells Using Non-Integrating Episomal Vectors and Feeder-Free Conditions

Aisha Mohamed [1,2], Theresa Chow [1,2], Jennifer Whiteley [1], Amanda Fantin [1,2], Kersti Sorra [2], Ryan Hicks [4] and Ian M. Rogers [1,2,3,5,*]

1 Lunenfeld Tanenbaum Research Institute, Sinai Health System, Toronto, M5G 1X5, Canada; aisha@lunenfeld.ca (A.M.); theresa.chow@mail.utoronto.ca (T.C.); jwhiteley@Lunenfeld.ca (J.W.); a.fantin@mail.utoronto.ca (A.F.)
2 Department of Physiology, University of Toronto, Toronto, M5S 1A8, Canada; kersti.sorra@mail.utoronto.ca
3 Soham & Shaila Ajmera Family Transplant Centre, University Health Network, Toronto, M5G 2C4, Canada
4 BioPharmaceuticals R&D Cell Therapy Department, Research and Early Development, Cardiovascular, Renal and Metabolism (CVRM), BioPharmaceuticals R&D, AstraZeneca, 431 83 Mölndal, Gothenburg, Sweden; Ryan.Hicks@astrazeneca.com
5 Division of Reproductive Sciences, Department of Obstetrics and Gynecology University of Toronto, Toronto, M5G 1E2, Canada
* Correspondence: rogers@lunenfeld.ca

Supplementary Figures

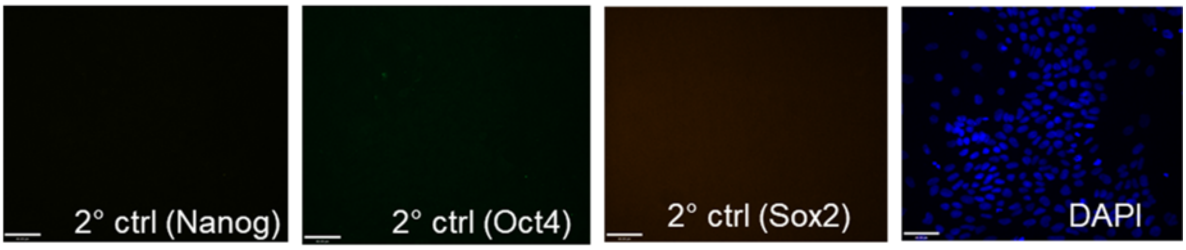

Figure S1. Secondary Antibody Controls. All secondary antibodies used did not produce any signal or cross reaction when used alone, without primary antibody. Examples for NANOG, OCT4 and SOX2. Scale bar = 60μm.

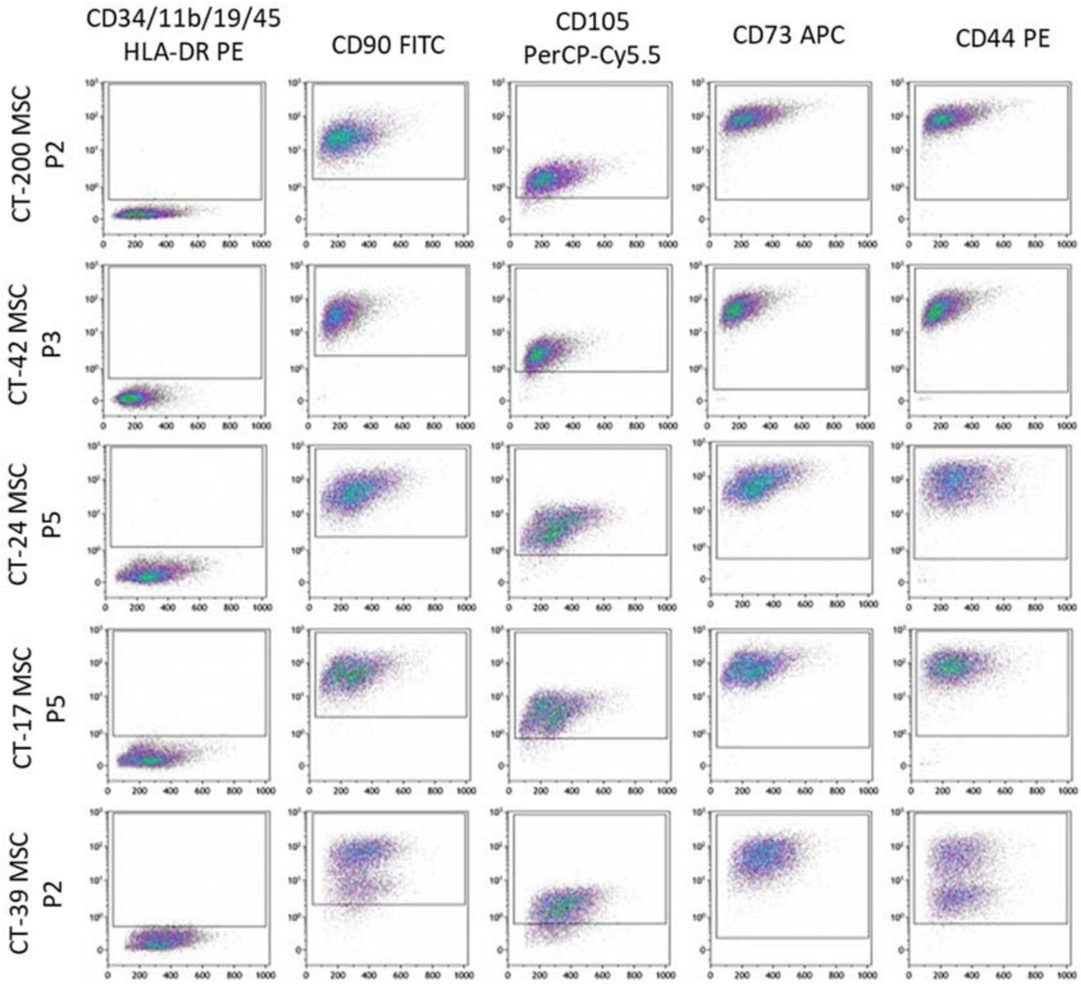

Figure S2. Flow cytometry dot plots for five of the CT-MSC lines used for reprogramming. Flow cytometry dot plots of MSCs cell surface markers CD44, CD73, CD90 and CD105 are positive and hematopoietic markers CD34, CD11b, CD19, HLA-DR and CD45 are negative (Y-axis=Fluorescence intensity, X axis= Side Scatter).

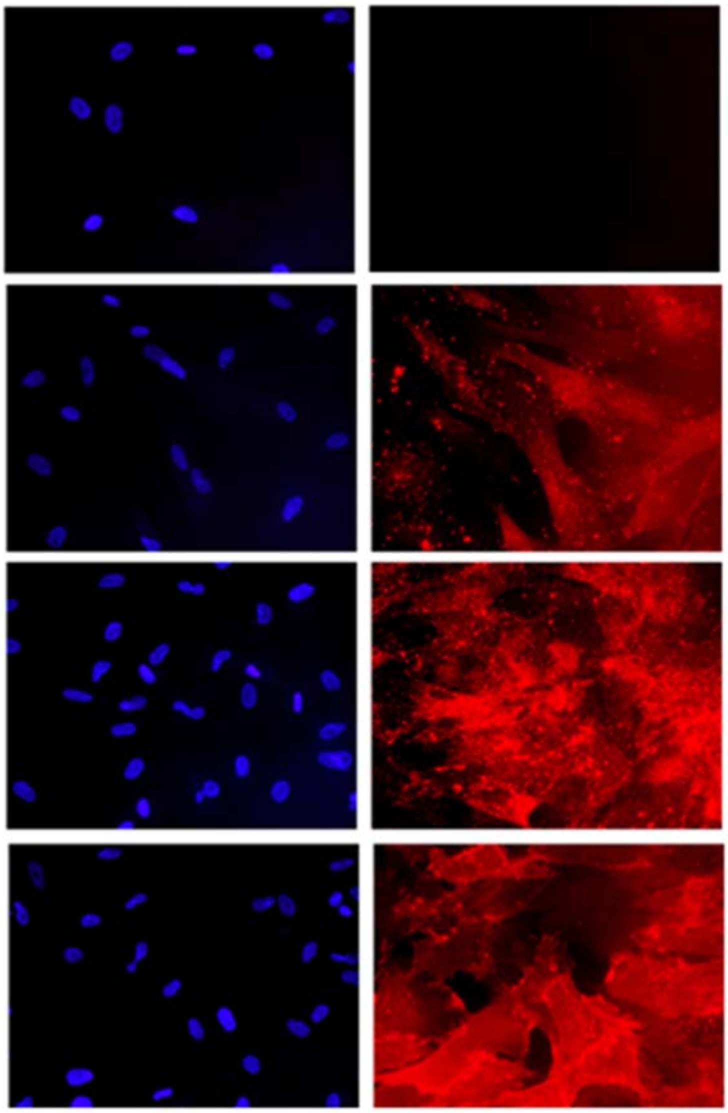

Figure 3. CT-MSC are negative for endothelial cells. CT-200 was passaged for five doublings (p4) in serum free media (Irvine Scientific, USA) and analyzed by ICC for mesenchymal cell proteins: CD73, CD90, Vimentin and for endothelial protein CD31 to determine if the media supported endothelial cell growth. There were no CD31+ endothelial cells.

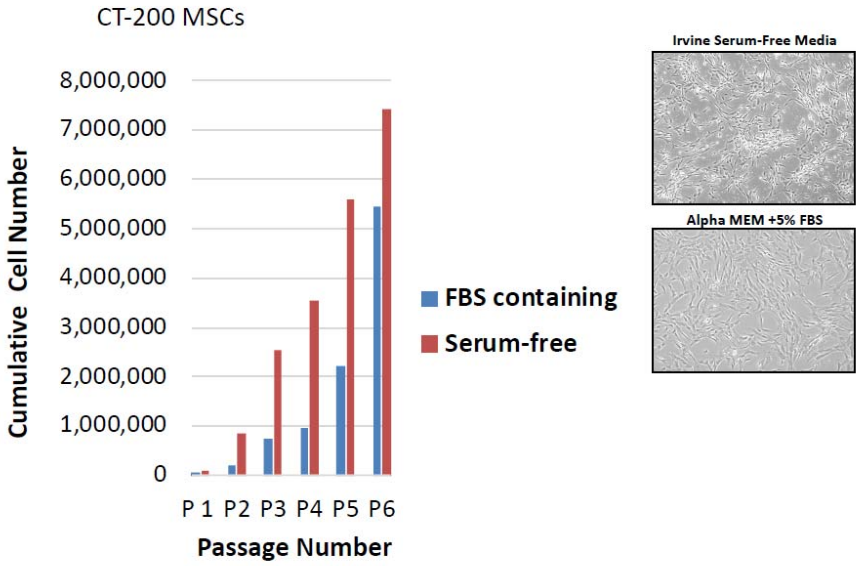

Figure S4. CT-MSC proliferate more in serum free media versus serum containing media. CT-MSC had a greater rate of proliferation in serum free (Irvine Scientific, USA) versus serum containing media.
